# Production of *Dichostereum sordulentum* Laccase and Its Entrapment in Lignocellulosic Biopolymers for Estrogen Biodegradation

**DOI:** 10.3390/molecules30244713

**Published:** 2025-12-09

**Authors:** Valeria Vázquez, Emiliana Botto, Alejandra Bertone, Marta Turull, Lúcia H. M. L. M. Santos, Victoria Giorgi, Fernando Bonfiglio, Javier García-Alonso, Pilar Menéndez, Karen Ovsejevi, Larissa Gioia

**Affiliations:** 1 Departamento de Biociencias, Facultad de Química, Universidad de la República, Montevideo 11800, Uruguay; valeria.svazquezp@gmail.com; 2 Departamento de Química Orgánica, Facultad de Química, Universidad de la República, Montevideo 11800, Uruguay; emilianabotto@fq.edu.uy (E.B.); vgiorgi@fq.edu.uy (V.G.); menendez@fq.edu.uy (P.M.); 3 Departamento de Ecología y Gestión Ambiental, Centro Universitario Región Este, Universidad de la República, Maldonado 20000, Uruguay; aleber77@hotmail.com (A.B.); jgalonso@cure.edu.uy (J.G.-A.); 4 Catalan Institute for Water Research (ICRA-CERCA), 17003 Girona, Spain; martaturull90@gmail.com (M.T.); luciahelena.santos@sapo.pt (L.H.M.L.M.S.); 5 Centro de Investigaciones en Biocombustibles 2G, Latitud—Fundación LATU, Montevideo 11500, Uruguay; fbonfig@latitud.org.uy

**Keywords:** laccase, semi-solid-state fermentation, enzyme immobilization, lignocellulosic hydrogel, 17α-ethinylestradiol, estrogen biodegradation

## Abstract

The widespread presence of estrogenic pollutants in aquatic environments poses a significant threat to ecosystems and human health, necessitating the development of efficient and sustainable removal technologies. This study aimed to develop a cost-effective biocatalyst for estrogen biodegradation using a fungal laccase. The enzyme was produced by the native strain *Dichostereum sordulentum* under semi-solid-state fermentation conditions optimized using a statistical Design of Experiments. The design evaluated carbon sources (glucose/glycerol), nitrogen sources (peptone/urea), inoculum size, and *Eucalyptus dunnii* bark as a solid support/substrate. The resulting laccase was entrapped within a hydrogel made of lignocellulosic biopolymers derived from a second-generation bioethanol by-product. Maximum laccase production was achieved with a high concentration of peptone (12 g/L), a low amount of bark (below 2.8 g), 8.5 g/L glucose and 300 mg/flask of inoculum. The subsequent immobilized laccase achieved 98.8 ± 0.5% removal of ethinylestradiol, outperforming the soluble enzyme. Furthermore, the treatment reduced the estrogenic biological activity by more than 170-fold. These findings demonstrate that the developed biocatalyst not only valorizes an industrial by-product but also represents an effective and sustainable platform for mitigating hazardous estrogenic pollution in water.

## 1. Introduction

The presence of pollutants in the environment is a worldwide concern. Contaminants such as pesticides, heavy metals, polycyclic aromatic hydrocarbons, microplastics, pharmaceuticals, and endocrine disruptors enter aquatic ecosystems through agricultural runoff, industrial effluents, and domestic wastewater. These pollutants threaten ecosystem health and human well-being through their acute toxicity, capacity for bioaccumulation, and potential long-term effects [1,2]. Of particular concern are Endocrine Disrupting Compounds (EDCs) due to their ability to interfere with the endocrine system even at very low concentrations. These substances—natural or synthetic—have complex effects on wildlife and humans, including embryo deformation and mortality, impaired growth and reproduction, feminization and intersex conditions in fish, as well as increased risks of breast cancer in women, prostate cancer in men, and thyroid dysfunction [3]. Their mechanisms of action vary widely. Many EDCs act as hormone mimics: for example, bisphenol A functions as a partial estrogen receptor (ER) agonist, while the synthetic estrogen 17α-ethinylestradiol (EE2) acts as a high-potency ER agonist. This mechanism activates estrogen-responsive pathways, including the induction of vitellogenin in male fish [4]. Other EDCs block receptor activity, as reported for anti-androgenic pesticides such as vinclozolin [5]. Additionally, polychlorinated biphenyls can interfere with thyroid hormone signaling by binding to the thyroid hormone receptor [6]. Finally, some compounds disrupt the normal synthesis and degradation of natural hormones by altering the activity of key enzymes in these metabolic pathways [7].

EDCs are present in everyday products such as detergents, plastics, pesticides and cosmetics, and reach the environment mainly through wastewater treatment plants [8]. The European Commission has identified the estrogens 17β-estradiol (E2) and 17α-ethinylestradiol (EE2) as contaminants of high concern due to their potent endocrine-disrupting effects [3,9]. EE2, widely used in hormonal contraceptives, exhibits stronger estrogenic responses, degrades more slowly, and persists longer in aquatic environments than E2 [10]. Consequently, environmental risk thresholds for EE2 are very stringent. Reported predicted-no-effect concentrations (PNECs) range from approximately 0.1 ng/L to 0.01 ng/L, depending on species sensitivity (e.g., fish and other aquatic organisms) [11,12] and the European Commission has proposed environmental quality standards (EQS) as low as 0.035 ng/L [13]. Crucially, environmental levels frequently exceed these thresholds by several orders of magnitude. In surface waters EE2 concentrations typically range from a few ng/L to several tens of ng/L [3,14], including values up to 89 ng/L reported in the Bouregreg River (Morocco) [15]. In Uruguay, concentrations up to 45 µg/L have been reported in coastal lagoons [11,16]. In municipal wastewater, influent concentrations vary widely, often between 10 and 300 ng/L, with peaks up to 7.9 µg/L, while secondary-treatment effluents commonly retain 1–30 ng/L of EE2 [17,18,19,20]. Given the limitations of conventional wastewater treatment to fully remove these estrogenic compounds, advanced biodegradation strategies have emerged as a promising and sustainable alternative [21,22,23]. Enzymatic treatments in particular, offer high selectivity, operate under mild reaction conditions, rely on renewable resources, and can transform contaminants into less toxic or non-toxic compounds [24,25].

Among the enzymes with potential for estrogen removal, fungal laccases (benzenediol: oxygen oxidoreductases, EC1.10.3.2) are especially relevant due to their broad substrate range and oxidative capacity. These enzymes can degrade endocrine disruptors, including natural estrogens (estrone, estriol, E2) and the synthetic compound EE2 [22,26,27,28,29,30,31,32,33,34]. Laccase production from native microorganisms is particularly relevant, as it provides novel enzymes with unexplored catalytic potential. Moreover, ensuring adequate enzyme yields is essential for practical application, and the choice of the culture system is a decisive factor. Semi-solid-state fermentation (SSSF) uses an insoluble solid support, partially mimicking the natural growth conditions of filamentous fungi. This system offers advantages over submerged fermentation, including lower operating costs and the valorization of agro-industrial residues as sustainable supports [35,36].

Despite their versatility, laccases still face industrial limitations such as instability, difficulty in recovering the enzyme, and lack of reusability. Enzyme immobilization is therefore a widely applied strategy to overcome these drawbacks [37,38,39]. Entrapment within polymeric matrices is one of the simplest and most cost-effective immobilization strategies. In this context, lignocellulosic hydrogels represent a cost-effective and environmentally sustainable alternative [40,41]. Ionic liquids (ILs) play a central role in their synthesis, as these molten salts behave as green, non-volatile, and thermally stable solvents capable of dissolving lignin and related biopolymers [42].

In previous work we developed a hydrogel synthesized from biopolymers derived from a residue of second-generation (2G) bioethanol production, which is predominantly composed of lignin. The most abundant linkage in this biopolymer is β-O-4, making it the primary target for cleavage by ILs, thereby enabling solubilization. Hydrogen bonding between lignin and ILs is a key mechanism to this process; these bonds are stronger than those between the lignin monomers themselves, leading to the stretching and breakage of the lignin structure. The addition of water shifts the H-bonding preference towards IL-water interactions, weakening the bonds with lignin. This causes lignin to precipitate as its original H-bonds reform, a process that entraps the enzyme leading to the formation of an active hydrogel [43,44,45]. Using this approach, a lignocellulosic hydrogel capable of entrapping *Dichostereum sordulentum* (DS) laccase was obtained. Furthermore, the potential of DS laccase for removing EE2 in both soluble and immobilized forms was demonstrated [46].

Prior to this study, DS laccase had only been obtained through submerged fermentation [47]. Thus, exploring its production under SSSF represents a novel and potentially more efficient alternative. In the present work, this strategy was implemented using *Eucalyptus dunnii* bark as the support and applying the statistical Design of Experiment (DOE) approach to identify key culture variables influencing enzyme production. Then, the enzyme was entrapped in the lignocellulosic hydrogel and its catalytic efficiency for EE2 removal was quantitatively evaluated using a highly sensitive UHPLC–MS/MS method. Finally, the reduction in estrogenic activity of the treated solution was assessed through the YES bioassay, providing an integrated evaluation of the biocatalyst’s biotechnological potential.

## 2. Results and Discussion

### 2.1. Optimization of Culture Medium for Laccase Production

Three sequential experimental designs were performed. Employing statistically based experimental design methodologies offers significant advantages in process optimization, including reducing the number of assays, saving time and reagents, and enabling the analysis of both individual effects and interactions among independent variables [48].

**Figure 1:**
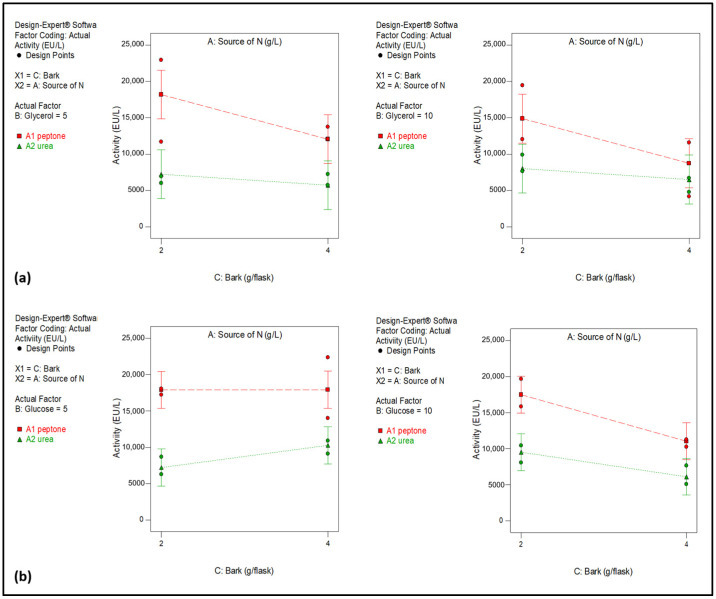
Multilevel categoric factorial design plot, showing the effect of N source, C source and bark on enzymatic activity for (**a**) glycerol and (**b**) glucose.

In the first study, the models for both carbon sources showed no significant lack of fit (Table 1 and Table 2), confirming their validity. Analysis of Variance (ANOVA) indicated that the nitrogen source was a significant factor in both cases. The results demonstrated that the highest laccase activity was obtained when peptone was used as the nitrogen source (Figure 1). Furthermore, when glucose was used as the carbon source, the interaction between glucose and bark was also significant (Figure 1a).

**Figure 2:**
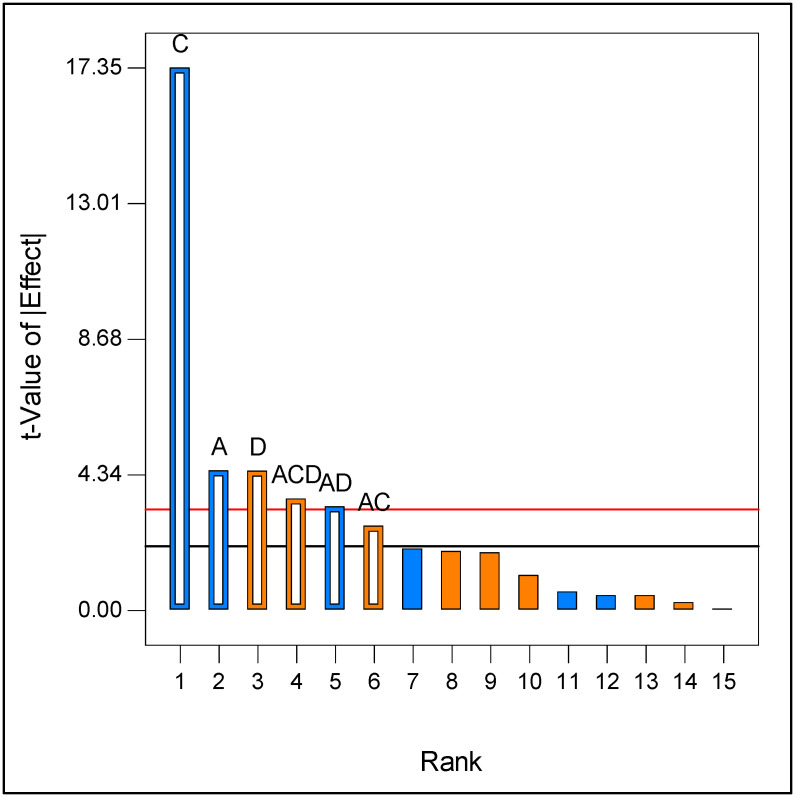
Pareto analysis chart (in orange factors with positive effects, in blue negative effects) for Full 2^4^ factorial design. A—Inoculum, C—Bark, D—Peptone. Bonferroni Limit 3.23131 (red line), *t*-Value Limit 2.05553 (black line).

Therefore, a full 2^4^ factorial design was then employed to study the effects of peptone concentration, glucose concentration, quantity of bark, and inoculum size. According to the Pareto chart (Figure 2), the amount of bark was the most negative factor, followed by the inoculum size. The concentration of peptone had a positive significant effect on the response, whereas the concentration of glucose showed no significant effect within the assessed range. Since the highest activity was obtained at the central points of the design (with a glucose concentration of 8.5 g/L), this concentration was selected for the subsequent study.

The model derived from this experiment was found to be significant (Table 3). Since the model presented curvature (for some factors there is no linear relationship with the response), it was appropriate to perform a Central Composite design to optimize the process.

**Figure 3:**
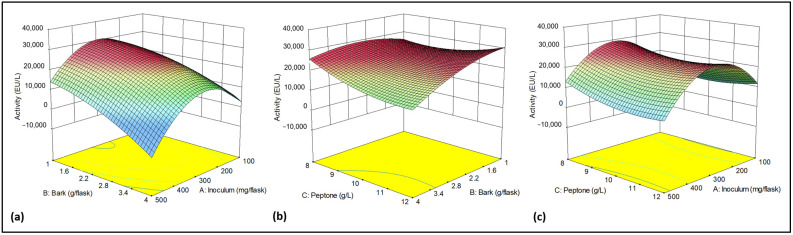
Three-dimensional graphs for Response Surface Quadratic model. (**a**) Effect of Bark and Inoculum (Peptone conc. = 12 g/L). (**b**) Effect of Bark and Peptone (Inoculum = 300 mg/flask). (**c**) Effect of Inoculum and Peptone (Bark = 2.5 g/flask). Activity (EU/L) decreases from red to sky blue.

The Central Composite design has several advantages, among them, it allows a second-order polynomial model to be fitted to the data. This model can capture nonlinear relationships between the input factors and the response. It provides enough information to estimate the main effects, the interactions, and the curvature of the response surface; moreover, it requires fewer experiments than other designs [49]. The analysis of variance revealed that the amount of bark was the most critical factor, with significant interactions observed between it and both the N source and the inoculum (Table 4). Plots in Figure 3 provide a visualization of the interaction between the studied factors and their influence on laccase activity. Activity increased as the bark content decreased, reaching a maximum between 1.0 and 2.8 g per flask, independent of peptone concentration, using an inoculum of 300 mg/flask. As it can be observed in Figure 3c, maximum enzyme production was achieved using 2.5 g of bark per flask and an inoculum of 300 mg/flask.

The resulting final equation for this model, in terms of actual factors was:

Activity (EU/L) = 24,975.94187 + 305.90068 × Inoculum + 21,489.68620 × Bark − 12,850.66832 × Peptone − 9.42430 × Inoculum × Bark − 4.09822 × Inoculum × Peptone − 1399.42989 × Bark × Peptone − 0.41247 × Inoculum^2^ − 1510.33822 × Bark^2^ + 830.79382 × Peptone^2^

The production of laccase by *D. sordulentum* had been reported only once before, in a study on ligninolytic enzyme production by a strain isolated in Uruguay [47]. In that work, a laccase activity of 6600 EU/L was achieved using a submerged fermentation system supplemented with *Eucalyptus* bark combined with CuSO_4_ as inducers. Notably, for the same strain in the present study, semi-solid-state fermentation (SSSF) successfully improved laccase production nearly fourfold.

It is well established that Cu^2+^ can act as a strong inducer of laccase expression, although its optimal concentration is highly species- and system-dependent [50]. In this work, CuSO_4_ was fixed at 1 mM to limit the number of variables in the DOE. This approach focused the experimental design on the major nutritional and process variables, but it also implies that the specific Cu^2+^ requirement of *D. sordulentum* under SSSF conditions remains to be determined. A dedicated optimization of copper concentration may therefore be valuable in future studies.

Beyond improving enzyme yields, this type of fermentation system also enables the valorization of a forestry residue through a culture method particularly well suited for the growth of filamentous fungi. *Eucalyptus* bark is generated in large quantities in Uruguay, where forestry is a major economic sector. Over one million hectares are dedicated to this industry, with *Eucalyptus* being the primary genus planted, and *E. dunnii* representing the second most important species, accounting for 25% of the forested area [51]. SSSF is a process based on solid-state culture with the addition of minimal free liquid to improve fermentation control and enhance fungal nutrient absorption [52,53]. In addition to allowing the use of a larger amount of residue, SSSF presents lower operational costs due to the absence of stirring requirements and yields an extract with higher enzymatic activity in a reduced volume compared to the submerged fermentation system.

### 2.2. Evaluation of EE2 Removal by Soluble and Immobilized Laccase

The efficiency of EE2 removal was quantitatively compared between the soluble and immobilized forms of *D. sordulentum* laccase using UHPLC-MS/MS. This technique was selected for its superior sensitivity and low limits of detection (26.1–27.3 ng/L for EE2 [54,55]), essential for the accurate quantification of trace-level contaminants [56].

For the soluble enzyme, a clear dose-dependent response was observed (Table 5). Increasing the enzyme units from 0.1 to 0.3 significantly enhanced EE2 removal, but a further increase to 0.5 EU yielded only marginal improvement. This plateau suggests product-mediated inhibition, where reactive intermediates (e.g., free radicals, quinoid, or polymerized compounds) may interfere with laccase activity at higher conversion levels [57,58].

In contrast, the immobilized biocatalyst proved significantly more effective, achieving 98.8 ± 0.5% EE2 removal. This result not only confirms but also quantitatively validates our previous findings obtained with HPLC-UV, where we reported ~97% removal [46].

Furthermore, it was confirmed that DS laccase can efficiently remove EE2 without a redox mediator, an additive typically required in many laccase-catalyzed processes to shuttle electrons and enable the oxidation of compounds that are not direct laccase substrates [59].

To elucidate the removal mechanism, the contribution of EE2 adsorption by the hydrogel matrix was investigated. The blank sample (B-EE2, hydrogels without entrapped enzyme) showed detectable decrease in EE2 concentration, suggesting analyte adsorption. This was confirmed by washing the hydrogels with AcOEt: from active hydrogels, 10.6 ± 5.7% of initial EE2 was recovered, versus ~20% from non-active hydrogels (B-EE2 W). These results demonstrate that while the blank hydrogel retains EE2 mainly through adsorption, the active hydrogel combines adsorption with enzymatic degradation, achieving more complete contaminant removal.

The demonstrated adsorptive capacity of the lignocellulosic matrix for EE2, also provides a relevant context for discussing the nature of the enzyme-support interaction. Covalent attachment is unlikely in this system, since effective covalent immobilization typically requires prior activation of either the enzyme or the support with specific reactive groups, as shown for lignocellulosic carriers in the study of Bezerra et al. [60]. Moreover, dissolution of the biopolymers in ionic liquids does not introduce new functional groups capable of forming covalent bonds with the enzyme. Instead, previous reports on laccase immobilization onto lignocellulosic materials (e.g., Posilipo et al. [61]) describe adsorption-based mechanisms. Given the composition of our support with a high lignin content [46], the absence of activating chemistry, and the conditions of entrapment, the most plausible interactions include ionic and hydrophobic forces, together with hydrogen bonding.

The distinct EE2 recoveries obtained for the active and blank hydrogels were consistent with their respective capacities to reduce estrogenic activity, as quantified by the Yeast Estrogen Screen (YES) assay (Table 6). This bioassay employs a genetically engineered yeast strain expressing the human estrogen receptor (hER), in which exposure to estrogenic compounds induces β-galactosidase production, detected via CPRG substrate conversion [62]. As expected from the literature, the synthetic compound EE2 showed a significantly higher estrogenic response compared to the natural hormone E2.

Statistical analysis (one-way ANOVA, F = 344.2841, *p* < 1.161 × 10^−10^) revealed significant differences among treatments, and Tukey HSD post hoc analysis confirmed that the active hydrogel (R-ILAC) exhibited a significantly higher EC_50_ than all other samples, which indicates lower estrogenic potency. Based on EC_50_ values, treatment with the active hydrogel beads reduced the estrogenicity of the solution by approximately 170-fold relative to untreated EE2, with a corresponding reduction in Relative Potency exceeding 99%. This effect significantly surpassed the approximately 10-fold decreases achieved by the blank hydrogel (B-EE2) and free enzyme (R-SLac) controls. These results highlight the clear advantage provided by enzyme immobilization within the lignocellulosic matrix.

Together, the UHPLC-MS/MS and YES results demonstrate that the immobilized biocatalyst not only removes EE2 but also drastically reduces the hormonal activity of the resulting transformation products, suggesting they show substantially lower affinity for the estrogen receptor. When compared with previous studies combining chemical degradation with biological validation, our system—achieving >99% reduction in estrogenic potency—stands among the most effective enzymatic strategies reported to date for estrogen detoxification [23,26]. The substantial reduction in estrogenic activity is a promising result concerning the safety of the biodegradation products. However, since non-estrogenic toxicity mechanisms could also occur, future work should include the identification of transformation products and the evaluation of additional toxicological endpoints, such as acute and chronic ecotoxicity in different model aquatic organisms [63]. This comprehensive approach is essential to fully evaluate the environmental safety and potential secondary effects of the treatment process.

Given that the hydrogel removes a fraction of EE2 not only through enzymatic degradation but also by adsorption, the need for environmentally safe post-use management of the spent hydrogel remains a critical consideration. Several handling options can be implemented, depending on regulatory requirements as well as technical and economic feasibility. These include safe disposal through incineration or landfilling with appropriate safeguards, and controlled biodegradation in systems designed to handle organic matrices containing trace-level pollutants. If material reuse or regeneration is pursued, it is essential to ensure that any residual adsorbed contaminant is either eliminated beforehand or effectively retained to prevent release during subsequent use [64].

## 3. Materials and Methods

### 3.1. Chemicals

1-Butyl-3-methylimidazolium acetate (BmimAc) was purchased from IoLi-Tec Ionic Liquids Technologies GmbH, Heilbronn, Germany. Ethinylestradiol and 2,6-dimethoxyphenol (DMP) were purchased from Sigma-Aldrich, St. Louis, MO, USA, and dimethyl sulfoxide from Carlo Erba (Milan, Italy).

### 3.2. Laccase Activity Assay

Laccase activity was determined using 2.0 mM DMP in 0.1 M sodium acetate buffer pH 3.8. The reaction was monitored by measuring the formation of quinone at 477 nm (ε_477_ = 14.800 M^−1^ cm^−1^) using a UV-1800 spectrophotometer, Shimadzu Corporation, Tokio, Japon. The reaction mixture was composed of 500 μL of DMP solution and 50 μL of laccase sample, prepared in the same buffer. One enzyme unit (EU) was defined as the amount of enzyme that catalyzed the appearance of 1 μmol of product per minute at 25 °C [46].

The immobilized enzyme activity was assayed by incubating active hydrogel beads with substrate under magnetic stirring (100 rpm). Supernatant aliquots were withdrawn from the reaction mixture at 30 s intervals and were returned afterward, keeping its volume unchanged. To detect enzyme release, after measuring the immobilized activity, the hydrogels beads were removed, the filtrates incubated at room temperature, and variation in absorbance at 477 nm determined again.

### 3.3. Protein Determination

Protein content was quantified using the bicinchoninic acid (BCA) assay, bovine serum albumin was used as a standard [65]. The amount of immobilized protein was calculated as the difference between the total protein applied to the gel and the protein recovered in the collected supernatants and washes.

### 3.4. Laccase Production

A native strain of *Dichostereum sordulentum* (1488), isolated from *Eucalyptus* forests in Uruguay [66], was employed for laccase production under semi-solid-state fermentation (SSSF) conditions. It was grown in potato dextrose agar (PDA) at 28 °C for 7 days. A preculture in liquid medium was made in cotton-plugged Erlenmeyer flasks (250 mL) containing 100 mL of malt extract 5%, Bactopeptone 1% and *Eucalyptus dunnii* bark 0.25%. It was inoculated with five agar plugs (10 mm diameter) from PDA culture and incubated at 28 °C on a rotary shaker at 150 rpm for 7 days. At the end of the incubation, the preculture was homogenized and centrifuged, giving a fungal biomass pellet. Unless otherwise indicated, the inoculum was prepared by resuspending 100 mg of the pellet in 3.0 mL of sterile 0.1 M sodium phosphate buffer pH 6.0. The basal medium consisted of CuSO_4_ 1 mM, KH_2_PO_4_ 2 g/L, MgSO_4_.7H_2_O 0.5 g/L, CaCl_2_.2H_2_O 0.1 g/L, in citrate-phosphate buffer 0.1 M, pH 5.0. *Eucalyptus dunii* bark was employed as the support-substrate (average granulometry 2 mm).

The concentration of CuSO_4_ was selected based on previous work with the same strain, where copper supplementation enhanced laccase expression [47], and on reports in the literature describing its inductive effect in basidiomycetes [50,52,67]. The concentration was kept constant to maintain a manageable number of factors within the DOE. The experiments for culture optimization were carried out in 250 mL flasks with 20 mL of liquid basal medium. They were incubated statically at 28 °C for 14 days. After that, 5 mL of 0.1 M pH 6.0 sodium phosphate buffer was added, shaken for 15 min, and centrifuged at 10,000 rpm for 10 min at 4 °C, laccase activity was measured in the supernatant.

### 3.5. Optimisation of Culture Medium for Laccase Production

Laccase production was optimized through a rational sequence of three experimental designs using the software Design Expert^®^ version 10.0 by Stat-Ease, Inc. (Suite 480, Minneapolis, MN, USA) for the design and analysis. The figures corresponding to the experimental designs were also generated using this software. Laccase activity measured in the culture supernatant was the monitored response, expressed in EU/L. Raw experimental data for all the experimental designs are shown in the Supporting Information.

#### 3.5.1. Multilevel Categoric Model

This statistical model was used to evaluate the effect on laccase production of nitrogen (N) source (peptone or urea); carbon (C) source (glycerol or glucose) and bark. For the N source, a fixed concentration (10 g/L) was used while C source concentration and bark amount varied between 5 and 10 g/L and 2 or 4 g per flask, respectively. These factors and ranges in which they were used were chosen based on literature and previous exploratory experiments. Two identical experimental designs were carried out, one for glucose and the other for glycerol, each one with 16 runs. The basal medium was supplemented with the C source, the N source and with *E. dunii* bark, as specified for each run in the design (see Table S1a,b in Supplementary Material). In the Analysis of variance values of “Prob > F” less than 0.0500 indicate model terms are significant. The residual error was decomposed in a pure error term and in a lack of fit term. The pure error was obtained from the replicates of experimental points.

#### 3.5.2. Full 2^4^ Factorial Design

Based on the results achieved with the Multilevel categoric model, Full 2^4^ factorial design was chosen to better adjust the levels of selected factors and assess a new factor to enhance laccase production. The new conditions were inoculum (100–1000 mg/flask), glucose (2–15 g/L), bark (1–5 g/flask) and peptone (2–10 g/L). Four central points were used, and runs were performed in duplicate, so the new design required 36 runs. The complete dataset for this design is provided in the Supplementary Material, Table S2.

#### 3.5.3. Central Composite Design

In this statistical design the levels of the factors that showed a negative effect in the Factorial design were decreased: inoculum (100–500 mg/flask), bark (1–4 g/flask). On the other hand, peptone concentration was increased (8–12 g/L) and glucose concentration was set at 8.5 g/L. The model included 5 central points generating a design of 19 runs, the raw data are provided in Table S3.

### 3.6. Active Hydrogel Formation

Active hydrogel was prepared under conditions previously reported. The starting materials were biopolymers obtained from the solid residue of *Eucalyptus globulus* biomass generated during bioethanol production, which had the following composition (*w*/*w*): 83.5 ± 0.3% lignin, 8.1 ± 0.3% cellulose, and 2.4 ± 0.1% hemicellulose [46].

One gram of BmimAc was placed in a 20 mL vial and heated up to 100 °C under gentle stirring with 0.6 mL of DMSO. Then, the lignocellulosic residue (175 mg) was added and stirred until dissolution. The mixture was cooled down to 40 °C, the lyophilized enzyme was added (30 EU), and the vial content was quickly transferred to a plastic syringe and dripped over 0.05 M pH 5.0 acetate buffer, giving beads of hydrogel with entrapped laccase. After removing the supernatant by filtration, the hydrogel beads were washed with the same buffer under gentle stirring conditions. Laccase activity was measured in supernatant, washes, and beads. The enzymatic activity retained after immobilization was 5 EU/g biopolymer.

### 3.7. Estrogen Biodegradation

Soluble laccase (SLac): A reaction mix (final volume = 5 mL) was prepared in 0.1 M sodium acetate buffer pH 5.0 containing aliquots of laccase (0.1, 0.3 and 0.5 EU) and ethinylestradiol (0.01 mg/mL). The mixture was gently agitated at 20 °C for 24 h and the reaction was stopped by freezing the samples, followed by lyophilization. Immobilized laccase (ILac): Suction-dried aliquots of insoluble enzyme, containing 0.1 EU, were incubated with EE2, following the same protocol as for the soluble enzyme, except that the reaction was stopped by removing the hydrogel by filtration. Three blanks were analyzed: soluble laccase in buffer solution, supernatant from the incubated suspension of active hydrogel in buffer, and supernatant from the incubated suspension of hydrogel beads without enzyme in EE2 solution. Once the hydrogels (with or without enzyme) were separated from the supernatants, they were washed with ethyl acetate and these washes were also analysed.

Analytical: Samples were analyzed on an Acquity Ultra-Performance^TM^ liquid chromatography system (Waters, Milford, MA, USA) coupled to a 5500 QTRAP hybrid triple quadrupole-linear ion trap mass spectrometry detector (Applied Biosystems, Foster City, CA, USA) (UHPLC-MS/MS), according to [55]. Lyophilized samples were redissolved in methanol (EE2, 25 mg/L) and centrifugated at 10,000 rpm during 10 min, giving the stock solutions. Aliquots of these solutions were diluted to achieve an EE2 concentration of 25 μg/L. 10 µL of a 1 mg/L standard solution of ethinylestradiol-d4 in methanol were added to the samples to obtain a final concentration of 10 µg/L. The samples were injected in the UHPLC-MS/MS system, under negative ionization mode, together with a calibration curve of analyte concentrations 0.52, 1.03, 5.15, 10.31, 25.77, 51.53 and 103.07 μg/L. The results were processed in the Analyst 1.5.1 software.

### 3.8. Yeast Estrogen Screen (YES) Bioassay

To determine the effectiveness of laccase enzyme on estrogenicity reduction by degradation of EE2, a yeast strain kindly provided by Prof. Eduard Routledge (Brunel University) was used, and the assay developed in laboratories of CURE Maldonado. The assay procedure was according to the original protocol [68] following the adaptations of Bila et al. [69]. Briefly, the yeast stock stored at −20 °C in a cryogenic tube (2 mL) with growth medium and glycerol (40%) was added to 10 mL of the growth medium and grew on an orbital shaker 48 h. 100 μL of culture were added for a new growth medium (10 mL) and grew on an orbital shaker for another 24 h. The assay medium was prepared by mixing 25 µL of the above solution, 25 mL of growth medium, and 250 µL of the Chromogenic substrate chlorophenol red-β-D-galactopyranoside (CPRG, 10 mg/mL). The 17β-estradiol (E2) standard solution (54.48 μg/L) and the samples extracts were serially diluted in ethanol and 10 µL of each dilution were transferred (in duplicate) into a 96-well cell culture flat bottom microtiter plate (Cellstar, Greiner Bio-One GmbH, Kremsmünster, Austria) and allowed to evaporate until dryness under laminar flow cabinet. Then 200 µL of the assay medium were seeded into each well and dilution series of E2 were used as a calibration curve. Plates were sealed with breathable masking tape and vigorously shaken on a plate shaker for 2 min. Then plates were incubated in darkness at 30 °C during 72 h for colour development and absorbance was read at 540 nm on a plate reader FLUOstar Optima (BMG Labtech, Ortenberg, Germany). Estrogenic activity was calculated as E2 equivalents (E2-EQ) by interpolation from the E2 standard curves (ng/L). The Effective Concentration 50 (EC_50_) was obtained from the Hill’s equation calculated from the sigmoidal curve generated by the serial dilution of the samples using R software (version 4.1.1, R Foundation for Statistical Computing, Vienna, Austria) with the drc package (version 0.3-3). Relative Potency (RP) was calculated as:

RP = EC_50(E2)_/EC_50(test compound)_.

ANOVA analyses were performed in order to determine a reduction on the estrogenic activity.

## 4. Conclusions and Future Work

Laccase from *Dichostereum sordulentum* was successfully produced through semi-solid-state fermentation using *Eucalyptus dunnii* bark, achieving a fourfold increase in enzymatic activity compared to the previous submerged fermentation system. When coupled with our previously developed immobilization approach—entrapping the enzyme in a biopolymeric net derived from a lignocellulosic bioethanol residue—the resulting biocatalyst proved highly effective. It achieved near-complete removal of 17α-ethinylestradiol (EE2) at concentrations exceeding those typically found in contaminated water bodies and drastically reduced the estrogenic activity of the treated solution, supporting its potential applicability for wastewater treatment processes.

This integrated process, which valorizes agricultural and industrial by-products, aligns with green chemistry principles by utilizing renewable resources and minimizing waste. Therefore, this technology represents a promising and sustainable approach for wastewater treatment that could provide added value to both the forestry industry and ethanol biorefineries. Future studies should build upon these findings by evaluating biocatalyst performance in authentic wastewater matrices, and characterizing the degradation products formed during EE2 transformation. Additional work may also expand the toxicity assessment beyond estrogenicity and explore environmentally safe strategies for post-use hydrogel management.

## Figures and Tables

**Table 1:** ANOVA [Partial sum of squares—Type III], multilevel categoric factorial design for Glucose.

Source	Sum of Squares	df	Mean Square	F Value	*p*-Value Prob > F
Model	3.339 × 10^8^	6	5.566 × 10^7^	9.17	0.0021
A-Source of N	2.433 × 10^8^	1	2.433 × 10^8^	40.07	0.0001
B-Glucose	2.085 × 10^7^	1	2.085 × 10^7^	3.43	0.0969
C-Bark	1.150 × 10^7^	1	1.150 × 10^7^	1.89	0.2020
AB	7.373 × 10^6^	1	7.373 × 10^6^	1.21	0.2991
AC	9.213 × 10^6^	1	9.213 × 10^6^	1.52	0.2492
BC	4.172 × 10^7^	1	4.172 × 10^7^	6.87	0.0277
Residual	5.464 × 10^7^	9	6.071 × 10^6^		
Lack of Fit	1.141 × 10^6^	1	1.141 × 10^6^	0.17	0.6904
Pure Error	5.350 × 10^7^	8	6.688 × 10^6^		
Cor Total	3.886 × 10^8^	15			

**Table 2:** ANOVA [Partial sum of squares—Type III], multilevel categoric factorial design for Glycerol.

Source	Sum of Squares	df	Mean Square	F Value	*p*-Value Prob > F
Model	2.768 × 10^8^	5	5.535 × 10^7^	4.07	0.0283
A-Source of N	1.739 × 10^8^	1	1.739 × 10^8^	12.78	0.0051
B-Glycerol	6.395 × 10^6^	1	6.395 × 10^6^	0.47	0.5087
C-Bark	5.839 × 10^7^	1	5.839 × 10^7^	4.29	0.0652
AB	1.666 × 10^7^	1	1.666 × 10^7^	1.22	0.2945
AC	2.143 × 10^7^	1	2.143 × 10^7^	1.57	0.2382
Residual	1.361 × 10^8^	10	1.361 × 10^7^		
Lack of Fit	1.066 × 10^7^	2	5.328 × 10^6^	0.34	0.7218
Pure Error	1.255 × 10^8^	8	1.568 × 10^7^		
Cor Total	4.129 × 10^8^	15			

**Table 3:** ANOVA of the adjusted model for Full 2^4^ factorial design.

Source	Sum of Squares	df	Mean Square	F Value	*p*-Value Prob > F
Model	1.769 × 10^9^	6	2.948 × 10^8^	58.68	<0.0001
A-Inoculum	1.004 × 10^8^	1	1.004 × 10^8^	19.97	0.0001
C-Bark	1.513 × 10^9^	1	1.513 × 10^9^	301.04	<0.0001
D-Peptone	9.977 × 10^7^	1	9.977 × 10^7^	19.86	0.0002
AC	3.649 × 10^7^	1	3.649 × 10^7^	7.26	0.0124
AD	5.522 × 10^7^	1	5.522 × 10^7^	10.99	0.0028
ACD	6.394 × 10^7^	1	6.394 × 10^7^	12.73	0.0015
Curvature	1.057 × 10^9^	1	1.057 × 10^9^	210.28	<0.0001
Residual	1.256 × 10^8^	25	5.024 × 10^6^		
Lack of Fit	6.914 × 10^7^	9	7.682 × 10^6^	2.18	0.0837
Pure Error	5.647 × 10^7^	16	3.529 × 10^6^		
Cor Total	2.951 × 10^9^	32			

**Table 4:** ANOVA for Response Surface Quadratic model [Partial sum of squares—Type III].

Source	Squares	df	Square	Value	Prob > F
Model	8.302 × 10^8^	9	9.224 × 10^7^	49.49	0.0002
A-Inoculum	6.184 × 10^6^	1	6.184 × 10^6^	3.32	0.1282
B-Bark	8.426 × 10^7^	1	8.426 × 10^7^	45.20	0.0011
C-Peptone	1.538 × 10^7^	1	1.538 × 10^7^	8.25	0.0349
AB	3.291 × 10^7^	1	3.291 × 10^7^	17.66	0.0085
AC	1.107 × 10^7^	1	1.107 × 10^7^	5.94	0.0589
BC	7.258 × 10^7^	1	7.258 × 10^7^	38.94	0.0015
A^2^	2.574 × 10^7^	1	2.574 × 10^7^	13.81	0.0138
B^2^	1.661 × 10^6^	1	1.661 × 10^6^	0.89	0.3885
C^2^	1.081 × 10^6^	1	1.081 × 10^6^	0.58	0.4806
Residual	9.320 × 10^6^	5	1.864 × 10^6^		
Lack of Fit	7.317 × 10^6^	2	3.659 × 10^6^	5.48	0.0996
Pure Error	2.003 × 10^6^	3	6.675 × 10^5^		
Cor Total	8.395 × 10^8^	14			

**Table 5:** Results of the UHPLC-MS/MS analysis.

Sample	EE2 Recovered (%)
EE2	102.6 ± 7.1
R-SLac 0.1	27.2 ± 2.1
R-SLac 0.3	9.6 ± 1.0
R-SLac 0.5	7.1 ± 1.5
R-ILac	1.2 ± 0.5
B-EE2	3.0 ± 0.2
B-ILac	<LOD
SLac	<LOD
R-ILac W	10.6 ± 5.7
B-ILac W	<LOD
B-EE2 W	20.2 *

EE2 = Ethinylestradiol in buffer. R-SLac = Reaction with soluble laccase (0.1 to 0.5 EU). R-ILac = Reaction with immobilized laccase. B-EE2 = Blank of reaction using hydrogel beads without enzyme. B-ILac = Active hydrogel beads in buffer without EE2. SLac = Laccase in buffer (not fortified). R-ILac W = Washes with ethyl acetate of active hydrogels previously used for estrogen degradation. B-ILac W = Washes with ethyl acetate of active hydrogels. B-EE2 W = Washes with ethyl acetate of B-EE2. Error shown represents standard deviation. * Unique sample.

**Table 6:** Effective concentration 50 (EC_50_) of different treatment with three replicates (mean ± SD).

Sample	EC_50_ (µg/L)	Relative Potency
E2	0.047 ± 0.02	
EE2	0.028 ± 0.02	1.68
EE2 + soluble laccase (R-SLac 0.5)	0.391 ± 0.33	1.20 × 10^−1^
EE2 + hydrogel beads (B-EE2)	0.598 ± 0.13	7.90 × 10^−2^
EE2 + active hydrogel beads (R-ILac)	4.819 ± 0.22	9.75 × 10^−3^

## Data Availability

The original contributions presented in this study are included in the article/Supplementary Material. Further inquiries can be directed to the corresponding authors.

## Supplementary Materials

The following supporting information can be downloaded at: https://www.mdpi.com/article/10.3390/molecules30244713/s1, Table S1a: Multilevel categoric factorial design for glycerol; Table S1b: Multilevel categoric factorial design for glucose; Table S2: Full Factorial Design; Table S3: Central Composite Design.
