# Supplementary material for: Production of Dichostereum sordulentum Laccase and Its Entrapment in Lignocellulosic Biopolymers for Estrogen Biodegradation
Source: Molecules. 2025 Dec 9;30(24):4713. doi: 10.3390/molecules30244713 (PMC12735493; doi:10.3390/molecules30244713)
Supplement: Supplementary file 1 [file molecules-30-04713-s001.zip › molecules-3996008-supplementary.pdf]

Supplementary Material

Production of Dichostereum sordulentum Laccase and Its Entrapment in Lignocellulosic Biopolymers for Estrogen Biodegradation

Valeria Vázquez[1], Emiliana Botto[2], Alejandra Bertone[3], Marta Turull[4,5], Lúcia H. M. L. M. Santos [4,5], Victoria Giorgi[2], Fernando Bonfiglio[6], Javier García-Alonso[3], Pilar Menéndez[2], Karen Ovsejevi[1*], Larissa Gioia[1**]

1 Departamento de Biociencias, Facultad de Química, Universidad de la República. Montevideo, Uruguay.
2 Departamento de Química Orgánica, Facultad de Química, Universidad de la República. Montevideo, Uruguay.
3 Departamento de Ecología y Gestión Ambiental, Centro Universitario Región Este, Universidad de la República. Maldonado, Uruguay.
4 Catalan Institute for Water Research (ICRA-CERCA). Girona, Spain.
5 University of Girona. Girona, Spain.
6 Centro de Investigaciones en Biocombustibles 2G, Latitud—Fundación LATU. Montevideo, Uruguay.
*E-mail: * kovsejev@fq.edu.uy, ** lgioia@fq.edu.uy*.

Contents

Table S1a Multilevel categoric factorial design for glycerol

Table S1b Multilevel categoric factorial design for glucose

Table S2 Full Factorial Design

Table S3 Central Composite Design

Optimisation of culture medium for laccase production

Experimental data

Table S1a Multilevel categoric factorial design for glycerol

	Factor 1	Factor 2	Factor 3	Response 1
Run	A:Source of N	B:Glycerol	C:Bark	Activity
	g/L	g/L	g/flask	EU/L
1	urea	10	4	4773
2	urea	5	4	5730
3	urea	10	2	9898
4	peptone	10	4	4168
5	urea	5	4	7241
6	peptone	10	2	12035
7	urea	5	2	6006
8	peptone	10	2	19442
9	urea	5	2	6926
10	peptone	5	4	12079
11	peptone	5	4	13738
12	urea	10	4	6699
13	urea	10	2	7638
14	peptone	10	4	11575
15	peptone	5	2	11688
16	peptone	5	2	22935

Table S1b Multilevel categoric factorial design for glucose

	Factor 1	Factor 2	Factor 3	Response 1
Run	A:Source of N	B:Glucose	C:Bark	Activiity
	g/L	g/L	g/flask	EU/L
1	urea	5	2	8677
2	peptone	10	2	19648
3	peptone	10	2	15837
4	peptone	5	2	17206
5	peptone	10	4	11272
6	urea	5	4	9104
7	peptone	5	4	22343
8	peptone	5	4	13997
9	peptone	10	4	10259

10	urea	10	2	10434
11	urea	5	2	6282
12	urea	5	4	10890
13	peptone	5	2	18033
14	urea	10	2	8065
15	urea	10	4	5094
16	urea	10	4	7658

Table S2 Full Factorial Design

	Factor 1	Factor 2	Factor 3	Factor 4	Response 1
Run	A:Inoculum	B:Glucose	C:Bark	D:Peptone	Activity
	mg/flask	g/L	g/flask	g/L	EU/L
1	1000	15	1	2	15408
2	1000	15	5	10	3799
3	1000	2	5	2	1318
4	100	2	1	2	14288
5	550	8,5	3	6	*
6	1000	15	1	10	15429
7	1000	2	5	10	5844
8	1000	2	1	10	13728
9	100	15	1	10	26876
10	1000	15	1	2	18416
11	1000	2	1	2	13535
12	1000	2	1	2	17560
13	1000	15	5	2	1878
14	100	15	5	10	5522
15	100	2	5	10	6603
16	550	8,5	3	6	35583
17	1000	2	5	2	2524
18	550	8,5	3	6	*
19	1000	15	1	10	19837
20	100	2	5	2	3563
21	100	15	5	10	5258
22	100	15	1	2	18007
23	100	2	5	10	8767
24	100	2	5	2	4047
25	100	15	1	10	25859
26	1000	15	5	2	1916
27	1000	2	5	10	2346

28	100	15	5	2	3423
29	100	2	1	2	14261
30	1000	2	1	10	9708
31	100	15	1	2	19272
32	100	15	5	2	3154
33	550	8,5	3	6	34528
34	100	2	1	10	25552
35	1000	15	5	10	9240
36	100	2	1	10	*

*Outliers removed to better adjustment of the model.

Table S3 Central Composite Design

	Factor 1	Factor 2	Factor 3	Response 1
Run	A:Inoculum	B:Bark	C:Peptone	Activity
	mg/flask	g/flask	g/L	EU/L
1	400	2,5	10	*
2	500	4	8	6485
3	500	1	8	13255
4	300	2,5	10	24852
5	200	2,5	10	20326
6	100	1	12	14670
7	500	4	12	*
8	300	2,5	9	*
9	100	1	8	6388
10	500	1	12	14137
11	300	2,5	10	25175
12	300	2,5	10	23884
13	300	2,5	11	23706
14	300	3,25	10	22732
15	100	4	8	11770
16	300	2,5	10	23426
17	100	4	12	2416
18	300	2,5	10	*
19	300	1,75	10	23539

*Outliers removed to better adjustment of the model.
